# Effects of Sodium Acetate and Sodium Butyrate on the Volatile Compounds in Mare’s Milk Based on GC-IMS Analysis

**DOI:** 10.3390/ani15060844

**Published:** 2025-03-15

**Authors:** Shengchen Zheng, Jianwei Lin, Yong Chen, Changjiang Zang, Fan Yang, Jiahao Li, Xiaobin Li

**Affiliations:** College of Animal Science, Xinjiang Agricultural University, Urumqi 830052, China

**Keywords:** short-chain fatty acids, acetic acid, butyric acid, mare milk, flavor compounds

## Abstract

This study aims to explore the impact of adding sodium acetate and sodium butyrate on the composition, blood biochemical parameters, and volatile flavor compounds of lactating mares’ milk. By assessing the influence of these additives on milk flavor enhancement, the findings provide scientific evidence for optimizing flavor characteristics and offer new strategies for improving the sensory attributes of mare milk products. Eighteen lactating Yili mares were randomly assigned to three groups: a control group, a sodium acetate group (85 mg/kg·BM^−1^), and a sodium butyrate group (85 mg/kg·BM^−1^). The experiment lasted 90 days, with milk yield recorded on days 0, 30, 60, and 90. Milk samples were collected on day 60 (peak lactation) for compositional analysis, and GC-IMS was employed to identify and quantify volatile compounds. Additionally, blood samples were drawn from the jugular vein before morning feeding on day 60 using heparinized tubes to assess key biochemical markers, including glucose, triglycerides, total cholesterol, and urea. The results revealed the following findings: (1) Milk yield and composition: The addition of sodium acetate and sodium butyrate had no significant effect on milk yield. However, both treatment groups exhibited significantly or extremely significantly higher milk fat content compared to the control group, whereas milk protein and lactose levels remained largely unchanged. (2) Blood biochemical indicators: The sodium butyrate group showed an extremely significant increase in urea levels compared to the sodium acetate and control groups. Glucose levels in the sodium acetate group were also significantly higher than in the control group. Moreover, triglyceride levels were markedly elevated in the sodium butyrate group compared to the sodium acetate group, while total bilirubin concentrations were significantly higher in the sodium acetate group than in the control group. (3) Volatile compounds: The addition of these additives led to a significant increase in the diversity and concentration of volatile compounds in mare milk. Notably, esters, aldehydes, and ketones showed substantial enrichment in both treatment groups. The relative abundance of esters such as butyl acetate, L-lactic acid ethyl ester, 1-pentene-3-ol, pentanol, and 3-pentanone increased, alongside a significant rise in aldehydes and ketones, including 2-heptenal and 3-pentanone. In conclusion, sodium acetate and sodium butyrate enhance milk flavor by modulating milk composition and metabolic parameters, providing a scientific foundation for improving the quality of mare milk products.

## Simple Summary

This study examines the impact of sodium acetate and sodium butyrate supplementation on the compositional profile, blood biochemical parameters, and volatile flavor compounds of lactating mares’ milk. By employing comprehensive analytical techniques, including Gas Chromatography–Ion Mobility Spectrometry (GC-IMS), this study elucidates the effects of these additives on both the chemical composition and sensory attributes of the milk. The findings offer critical insights into optimizing its flavor profile, enhancing overall quality, market appeal, and nutritional value.

## 1. Introduction

Milk is not only a rich source of proteins, fats, lactose, and minerals but also contains distinctive flavor compounds that contribute to its nutritional value and sensory characteristics [1]. These flavor compounds, primarily aldehydes, ketones, esters, and other volatile substances, play a pivotal role in determining milk’s taste profile [2]. The concentration and composition of free fatty acids significantly influence flavor development, as fatty acids are absorbed from the bloodstream by mammary epithelial cells and synthesized within the mammary glands [3]. Previous studies have demonstrated the role of short-chain fatty acids (SCFAs) in modulating milk composition. Kong Qingyang et al. [4] reported that adding acetic acid, propionic acid, and butyric acid to dairy cows’ diets enhances triglyceride content in mammary epithelial cells while altering the composition and concentration of free fatty acids in milk. These fatty acids serve as both substrates for milk fat synthesis and regulatory factors in lipid metabolism, thereby influencing milk fat synthesis at the cellular level. Beyond their role in lipid metabolism, fatty acids also modulate the composition and diversity of flavor compounds in milk. Wang Yaling [5] found that dietary addition of acetic acid salts in dairy goats increased palmitic acid content in milk, enhancing its creaminess and overall flavor profile. Additionally, studies have established that acids, alcohols, and esters are the predominant flavor compounds in cow’s and goat’s milk, serving as key markers for differentiating milk from various sources [6]. Mare milk shares a similar nutritional composition with cow and goat milk, yet research has primarily focused on its amino acid and fatty acid profiles, with limited insights into its volatile flavor compounds and the mechanisms governing their formation. Given this gap, the present study investigates the effects of sodium acetate and sodium butyrate addition to the diet of mares on the types, composition, and relative abundance of flavor compounds in mares’ milk. The findings aim to provide a scientific basis for optimizing flavor profiles in mare milk products through targeted feeding strategies.

## 2. Materials and Methods

### 2.1. Ethical Considerations

All animal care and handling procedures adhered to the Guidance of the Care and Use of Laboratory Animals in China and were approved by the Animal Care Committee of Xinjiang Agricultural University (protocol permit number: 2020024).

### 2.2. Supplementation Agents

Sodium acetate (≥98%) and sodium butyrate (≥98%) were purchased from Xinjiang Zhimai Trading Co., Ltd, which is located in Urumqi, Xinjiang, China.

### 2.3. Experimental Animals

To reduce the impact of individual differences on the experimental results, we initially selected 50 Yili mares as experimental subjects. From these mares, 18 individuals with the most comparable characteristics—such as milk yield, composition, body weight, and body measurements—were chosen for the experiment. The selected mares were aged 5–8 years, had 2–4 parities, and exhibited an average body weight of 422.66 ± 37.28 kg, with a daily milk yield of 2.80 ± 0.41 kg at 30 days of lactation. Prior to the experiment, all mares underwent veterinary examinations to confirm their health status, ensuring the reliability and representativeness of the subsequent trials.

### 2.4. Experimental Design

The experiment was conducted from June to September 2023 at Zhaosu Horse Farm, located in the southwest of Yili Kazakh Autonomous Prefecture, Xinjiang (43°09′–43°15′ N, 80°08′–81°30′ E). The 18 mares were randomly assigned to three groups (control, sodium acetate, and sodium butyrate), with six individuals per group. All mares were maintained under identical grazing conditions, including standardized grazing time, water access, milking schedule, and pasture. Only the experimental groups received additives (85 mg/kg·BW/d·mare) using specialized feed bags, while the control group received no additives. The trial lasted 90 days, with milk yield recorded on days 0, 30, 60, and 90. On day 60 (peak lactation), milk samples were collected for compositional and volatile compound analysis. Additionally, blood samples were collected from the jugular vein in heparinized tubes before morning feeding on the same day.

### 2.5. Feeding Management

Mares followed an 8 h milking schedule during the day, during which foals, housed in pens, were separated to prevent milk consumption. The remaining 16 h allowed foals to graze alongside their mothers. At 09:00 daily, the experimental groups received their respective supplements via specialized feed bags, with mares separated from foals for milking. After the final milking at 17:00, mares and foals were reunited, allowing unrestricted nursing. Throughout the study, all mares had ad libitum access to forage and water. Pasture samples from grazing areas were collected on days 30, 60, and 90 to assess forage nutritional composition. The nutrient composition of the pasture is presented in Table 1.

### 2.6. Sample Collection

On the 60th experimental day, 5 mL of blood was drawn from the jugular vein before morning feeding using heparinized tubes. The samples were centrifuged at 3500 r/min for 15 min to isolate serum, which was subsequently stored at −20 °C for further analysis. Milk samples were collected at 11:00, 13:00, 15:00, and 17:00 post-addition, with 25 mL obtained at each time point. The samples were pooled, aliquoted into 5 mL cryotubes, and flash-frozen in liquid nitrogen for subsequent analysis.

### 2.7. Sample Analysis

#### 2.7.1. Milk Yield Measurement

Milking was conducted at 11:00, 13:00, 15:00, and 17:00 on days 0, 30, 60, and 90, with milk yield quantified using an electronic scale immediately after each session.

#### 2.7.2. Milk Composition Measurement

At each milking time point, 25 mL of milk was collected from the four samples, yielding a total of 100 mL, which was homogenized and analyzed for composition using a Lactoscan multifunctional milk analyzer. The Lactoscan multifunctional milk analyzer (Lactoscan SP) was sourced from Milkotronic Ltd., Nova Zagora, Bulgaria. Following calibration and 20 min of stabilization, the system was thoroughly flushed with distilled water before introducing the milk sample into the detection chamber. The concentrations of milk fat, protein, lactose, and non-fat solids were recorded.

#### 2.7.3. Blood Biochemical Indicators

All biochemical assays were conducted using an automatic biochemical analyzer (BS-240VET, Shenzhen Mindray Bio-Medical Electronics Co., Ltd., Shenzhen, China), with reagent kits procured from the same manufacturer. Assays were performed according to the kit instructions.

Blood nitrogen metabolism parameters included total protein, albumin, globulin, and urea, while glucose and lipid metabolism markers encompassed glucose, triglycerides, total bilirubin, direct bilirubin, and total cholesterol.

#### 2.7.4. Measurement of Volatile Compounds in Milk

Volatile compounds are low molecular weight chemical substances that can volatilize from mare milk into gas or vapor form under standard temperature and pressure conditions. Volatile compounds in mare milk were analyzed using the FlavourSpec^®^ Gas Chromatography–Ion Mobility Spectrometry (GC-IMS) system (G.A.S., Dortmund, Germany) in combination with the PAL3-RSI Series II static headspace autosampler (CTC Analytics AG, Zwingen, Switzerland). A 2 mL aliquot of each sample was incubated at 60 °C for 20 min before automated injection via static headspace technology. Each sample was analyzed in triplicate. The GC conditions were set to a column temperature of 60 °C, with high-purity nitrogen (99.999% purity) as the carrier gas, and a programmed flow ramp to ensure high-resolution separation and detection of volatile components. Ionization was achieved using a tritium source, with a 53 mm migration tube maintained at high voltage and optimized internal temperature. The n-ketones used in this study included 2-butanone, 2-pentanone, 2-hexanone, 2-heptanone, 2-octanone, and 2-nonanone, all of analytical grade and supplied by Shanghai Aladdin Biochemical Technology Co., Ltd., Shanghai, China. The standard gas used for sample processing was nitrogen (99.999% purity). Sample containers were 20 mL headspace vials supplied by Shandong Haineng Scientific Instrument Co., Ltd., Jinan, Shandong, China. The chromatographic column was an MXT-WAX capillary column (30 m × 0.53 mm, 1.0 μm) supplied by Restek Corporation, Bellefonte, PA, USA.

### 2.8. Data Processing

A mixed standard solution of six ketones was used to establish a calibration curve based on retention time and retention index. Retention indices were calculated using the retention times of target compounds, with qualitative identification performed by referencing the GC retention index database and the IMS migration time database.

Volatile compounds were visualized and compared using three-dimensional and two-dimensional spectra, differential spectra, fingerprint plots, and PCA graphs generated via the VOCal software’s Reporter, Gallery Plot, and Dynamic PCA plugins (version 0.4.03, G.A.S. Company, Dortmund, Germany).

Peak volume data were normalized in Excel and subjected to ANOVA using SPSS 19.0. Results are expressed as mean ± standard deviation, with statistical significance set at *p* < 0.05 and extreme significance at *p* < 0.01.

## 3. Results

### 3.1. Effects of Adding Sodium Acetate and Sodium Butyrate on Milk Yield in Mares

**Figure 1:**
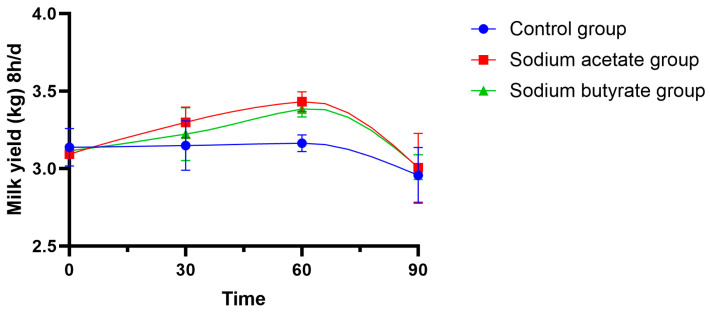
Trends of mare milk yield changes over 8 h after addition of sodium acetate and sodium butyrate.

The effects of adding sodium acetate and sodium butyrate on milk yield in mares are presented in Figure 1. While neither additive significantly affected milk yield, a trend toward increased production was observed compared to the control group, with sodium acetate exhibiting a more pronounced effect than sodium butyrate.

### 3.2. Effects of Adding Sodium Acetate and Sodium Butyrate on Milk Composition in Mares

As shown in Table 2, milk fat percentage significantly increased following addition, with sodium acetate leading to a 39.17% rise (*p* < 0.05) and sodium butyrate inducing a 50% increase (*p* < 0.01) compared to the control group. Although relative concentrations of milk fat, protein, lactose, and non-fat solids were higher in the added groups, these differences did not reach statistical significance (*p* > 0.05).

### 3.3. Effects of Adding Sodium Acetate and Sodium Butyrate on Blood Biochemical Indicators in Mares

Table 3 details the effects of sodium acetate and sodium butyrate addition on blood biochemical indices. Urea content in the sodium butyrate group was significantly higher than in sodium acetate and control groups, with increases of 19.96% and 23.46%, respectively (*p* < 0.01).

Glucose content in the sodium acetate group was significantly elevated compared to the control group (*p* < 0.01) and the sodium butyrate group (*p* < 0.05), rising by 90.26% and 50.81%, respectively.

Triglyceride content was markedly higher in the sodium butyrate group than in the sodium acetate group (*p* < 0.01), showing a 52.78% increase. Additionally, total bilirubin content was significantly elevated in the sodium acetate group compared to the control group (*p* < 0.01). Furthermore, total cholesterol content in the sodium acetate group exceeded those in the sodium butyrate group by 19.29% (*p* < 0.01).

### 3.4. Comparative Analysis of Volatile Compounds in Mare Milk

**Figure 2:**
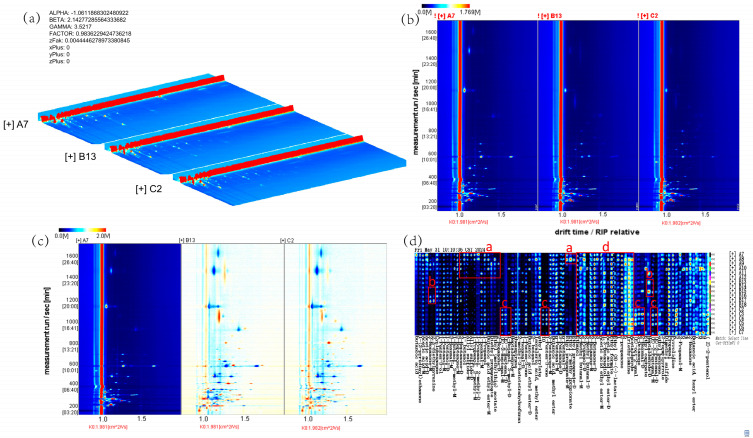
(**a**) Three-dimensional GC-IMS spectrum, (**b**) two-dimensional GC-IMS spectrum, (**c**) differential spectrum, and (**d**) fingerprint spectrum of volatile compounds in mare milk.

Figure 2a presents the three-dimensional GC-IMS spectrum, depicting the spatial distribution of volatile compounds in mare milk across the control, sodium acetate, and sodium butyrate groups. Figure 2b displays the two-dimensional spectrum, where differences in peak intensities reflect variations in volatile profiles among the groups. Figure 2c illustrates the differential spectrum, providing a more intuitive visualization of peak intensity disparities. Figure 2d shows the fingerprint spectrum, categorizing volatile compounds into four distinct regions: region a represents characteristic volatiles in mare milk without additives; region b and region c correspond to volatiles specifically enriched following sodium acetate and sodium butyrate addition, respectively; region d includes volatile compounds common to all three groups.

### 3.5. Cluster Analysis of Volatile Compounds

**Figure 3:**
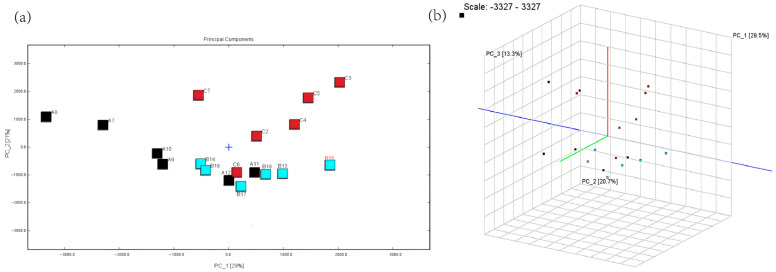
QPCA score plot of volatile components in mare milk: (**a**) PCA plot; (**b**) 3D PCA plot.

Principal component analysis (PCA) was performed to assess the volatile compounds in mare milk, as shown in Figure 3. Figure 3a illustrates that the first and second principal components (PC_1 and PC_2) explained 29.0% and 20.7% of the variance, respectively, accounting for a cumulative contribution of 50%. The control, sodium acetate, and sodium butyrate groups formed distinct clusters, indicating significant differences in their volatile compound profiles. Figure 3b presents a 3D PCA visualization, where data points, represented by colored squares, indicate three distinct groups; the blue, green, and black squares represent these groups. The lines corresponding to PC_1, PC_2, and PC_3 depict the directions of maximum variance, explaining 29.5%, 20.7%, and 13.3% of the variance, respectively.

### 3.6. Qualitative Analysis of Volatile Compounds in Mare Milk

**Figure 4:**
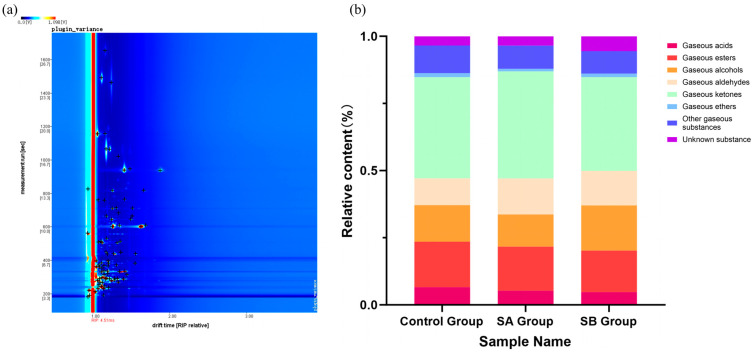
Qualitative GC-IMS spectrum of volatile components in mare milk and the relative proportions of various volatile substances. (**a**) Qualitative GC-IMS spectrum. (**b**) Relative proportions of various volatile substances.

Figure 4 shows the qualitative GC-IMS spectrum of volatile components in mare milk and the relative proportions of various volatile substances. The figure shows that 86 volatile compounds, including monomers and dimers, were detected when the GC-IMS database was used for compound identification. These included 16 esters, three acids, 15 alcohols, 15 ketones, 18 aldehydes, nine other compounds, and 10 unidentified compounds.

### 3.7. Qualitative and Quantitative Analysis of Volatile Compounds in Mare Milk

#### 3.7.1. Changes in Volatile Acid Ions in Mare Milk

As shown in Table 4, three volatile acid ions were detected: butyric acid, acetic acid-M, and acetic acid-D. The relative abundances of acetic acid-M and acetic acid-D were significantly lower in the sodium butyrate group compared to the control group (*p* < 0.01). Similarly, the relative content of acetic acid-D in the sodium acetate group was significantly reduced compared to the control group (*p* < 0.01).

#### 3.7.2. Changes in Volatile Ester Ions in Mare Milk

As presented in Table 5, 16 volatile ester ions were identified, with notable changes observed [7] in diethyl malonate, sec-butyl acetate, ethyl hexanoate, and sec-butyl acetate. The sodium acetate and sodium butyrate groups exhibited elevated relative contents of butyl acetate, L-lactate ethyl ester, and ethyl hexanoate compared to the control group, though these differences were not statistically significant (*p* > 0.05). However, the relative content of ethyl hexanoate was significantly higher in the sodium acetate group than in the control group (*p* < 0.05). Conversely, diethyl malonate levels were significantly lower in both added groups compared to the control (*p* < 0.05), while sec-butyl acetate was significantly reduced in the sodium butyrate group (*p* < 0.05).

#### 3.7.3. Changes in Volatile Alcohol Ions in Mare Milk

Table 6 details the 15 volatile alcohol ions detected, among which significant differences were noted for 1-pentene-3-ol, pentanol-M, butanol, ethanol, isopropanol-D, and cis-2-pentene-1-ol. In the sodium acetate group, 1-pentene-3-ol (*p* < 0.01), pentanol (*p* < 0.01), and isopropanol-D (*p* < 0.05) showed significantly or extremely significantly higher relative contents than in the control group. In the sodium butyrate group, the relative levels of 1-pentene-3-ol (*p* < 0.05), butanol (*p* < 0.01), cis-2-pentene-1-ol (*p* < 0.01), and ethanol (*p* < 0.05) were significantly or extremely significantly increased compared to the control. Additionally, the sodium butyrate group exhibited significantly higher relative contents of butanol (*p* < 0.01), cis-2-pentene-1-ol (*p* < 0.01), and ethanol (*p* < 0.05) compared to the sodium acetate group.

#### 3.7.4. Changes in Volatile Aldehyde Ions in Mare Milk

As shown in Table 7, 18 volatile aldehyde ions were detected, with significant changes in 2-hexenal-M, hexanal-M, 2-heptenal-D, 2-heptenal-M, heptanal, pentanal, 3-methylbutanal-M, 2-hexenal-D, heptanal-D, and propanal. In the sodium butyrate group, the relative abundances of 2-hexenal-M, hexanal-M, and 2-heptenal-M-D were significantly higher than in both the control and sodium acetate groups (*p* < 0.01). Additionally, heptanal-M, pentanal-D, and 3-methylbutanal-M levels were significantly elevated compared to the control group (*p* < 0.05). The sodium acetate group exhibited significantly higher levels of 2-hexenal-D and heptanal-D than the control (*p* < 0.05). Moreover, the sodium butyrate group displayed an extremely significant increase in propanal content relative to the control (*p* < 0.01) and a significant increase compared to the sodium acetate group (*p* < 0.05).

#### 3.7.5. Changes in Volatile Ketone Ions in Mare Milk

As detailed in Table 8, 15 volatile ketone ions were identified, with significant alterations in 2-heptanone-M, 2-nonanone-D, 2-methyl-tetrahydrofuran-3-one, 1-pentene-3-one-M, 3-pentanone-M, 3-pentanone-D, 2-butanone-M, and 2-hexanone. In the sodium butyrate group, the relative contents of 2-heptanone-M, 2-nonanone-D, 1-pentene-3-one-M, 3-pentanone-M, 3-pentanone-D, and 2-butanone-M were significantly elevated compared to the control (*p* < 0.01). Meanwhile, 2-methyl-tetrahydrofuran-3-one was significantly reduced relative to the control (*p* < 0.05), and 2-hexanone exhibited an extremely significant decrease (*p* < 0.01). Furthermore, 2-heptanone-M, 2-nonanone-D, 3-pentanone-M, and 3-pentanone-D levels were significantly higher in the sodium butyrate group than in the sodium acetate group (*p* < 0.01).

#### 3.7.6. Changes in Other Volatile Ions in Mare Milk

As summarized in Table 9, nine additional volatile ions were detected, with significant changes observed in allyl propyl sulfide-M, methyl sulfide, and 2-acetylpyridine. The sodium butyrate group exhibited significantly higher relative levels of all three compounds compared to the control (*p* < 0.01). Additionally, the relative abundance of methyl sulfide was significantly greater in the sodium butyrate group than in the sodium acetate group (*p* < 0.05). Conversely, the sodium acetate group showed a significant increase in allyl propyl sulfide-M compared to the control group (*p* < 0.05).

## 4. Discussion

SCFAs, defined as saturated fatty acids with six or fewer carbon atoms, are also referred to as volatile fatty acids due to their inherent volatility [8]. The primary SCFAs include acetic acid, propionic acid, butyric acid, and valeric acid. SCFAs originate from two major pathways: dietary intake of exogenous SCFAs, which are absorbed into the bloodstream and transported to various tissues [9], and microbial fermentation of carbohydrates in the gastrointestinal tract following carbohydrate consumption by animals [10,11]. In herbivores, microbial carbohydrate fermentation serves as the predominant source of SCFAs. In ruminants, the majority of SCFAs are synthesized via rumen fermentation, with a smaller fraction generated by intestinal microbiota. These SCFAs primarily exist in ionic form and are absorbed through the rumen or intestinal epithelium, subsequently entering different tissues to participate in various physiological processes, including energy metabolism and nutrient utilization [12]. In monogastric herbivores, anaerobic bacteria in the large intestines (cecum and colon) ferment undigested and unabsorbed carbohydrates, producing SCFAs that serve as key nutrients and primary energy substrates [13]. For instance, propionic acid acts as a key gluconeogenic precursor, contributing to energy metabolism via glucose synthesis, whereas acetic acid and butyric acid function as both substrates for de novo fatty acid synthesis in the mammary gland and signaling molecules regulating mammary fatty acid metabolism, thereby influencing milk fat composition and content [14]. Urrutia et al. [15] reported that supplementing dairy cow diets with 2.9% acetate significantly increased milk fat percentage and yield by 0.2% and 90 g/d, respectively, whereas an equimolar butyrate addition did not exert a significant effect on either parameter. Matamoros et al. [14] reported that dietary supplementation with 3.25% acetate significantly increased milk fat yield in dairy cows, likely due to enhanced acetate supply to the mammary glands, which stimulated de novo fatty acid synthesis. Studies on ruminal SCFA infusion in dairy cows further demonstrated that acetate and butyrate administration significantly elevated milk fat percentage, milk fat yield, and the concentration of de novo-synthesized fatty acids (<C16 and C16), particularly increasing palmitic acid content and yield. A clear dose–response relationship was observed between acetate infusion and milk fat synthesis, with both milk fat percentage and milk fat yield exhibiting linear and quadratic increases in response to acetate supply. These findings suggest that augmenting ruminal acetate and butyrate availability in lactating dairy cows enhances milk fat yield by providing essential substrates for de novo fatty acid synthesis through increased acetate and BHB levels [16]. In the present study, sodium acetate and sodium butyrate addition over an 8 h period led to an increase in milk yield by lactating mares, as well as higher milk protein, fat, and lactose contents. Notably, sodium acetate addition significantly elevated milk fat content, whereas sodium butyrate addition significantly increased milk protein levels. These findings align with the results reported by Urrutia et al. [13] and Matamoros et al. [14] in dairy cows. The capacity of acetic acid and butyric acid to enhance milk fat content is attributed not only to their role as metabolic energy sources and precursors for de novo fatty acid synthesis but also to their regulatory effects on the expression of mammary gland genes involved in fatty acid metabolism. Sun et al. [17] demonstrated that butyric acid and propionic acid promoted triacylglycerol accumulation and lipid droplet formation in goat mammary epithelial cells while concurrently upregulating key genes associated with fatty acid synthesis, including SCD1, FABP3, and PPARG. These findings indicate that SCFAs improve milk fat composition and mammary gland function by enhancing the fatty acid biosynthesis pathway. Blood biochemical parameters serve as critical indicators of an organism’s health status, providing essential insights into metabolic activity and physiological functions. These parameters reflect key processes such as glucose metabolism, lipid metabolism, and nitrogen metabolism [18].

Carbohydrate metabolism encompasses glucose synthesis, utilization, and storage, directly influencing an organism’s energy balance and expenditure [19]. These metabolic processes affect not only growth and development but also overall metabolic efficiency, production performance, and health status in animals [20]. Blood glucose serves as a critical biomarker of energy homeostasis, reflecting an organism’s energy requirements and regulatory mechanisms. Its concentration is modulated by dietary intake, insulin secretion, and hepatic gluconeogenesis, impacting growth, metabolism, and nutrient utilization efficiency [21,22]. Fluctuations in blood glucose levels not only affect physiological homeostasis but also have direct implications for production performance and animal health [23]. In the present study, plasma glucose concentrations in mares supplemented with sodium acetate were significantly higher than in the control group and the sodium butyrate group. This finding is consistent with the results reported by Cheng Yating et al. [24] in sows, suggesting that SCFAs may enhance glucose metabolism by activating G protein-coupled receptors and stimulating insulin secretion, thereby increasing plasma glucose levels in the supplemented groups. Additionally, sodium acetate and sodium butyrate, as rapidly absorbed SCFAs, can be efficiently metabolized in the intestine and utilized as an immediate energy source. Their rapid conversion into ATP supports glucose synthesis and metabolism, improving overall energy homeostasis and contributing to elevated plasma glucose levels in mares [12].

Lipid metabolism encompasses the synthesis, storage, and breakdown of lipid molecules, including fatty acids, triglycerides, and cholesterol, playing a pivotal role in energy homeostasis, cell membrane integrity, and hormone biosynthesis [25]. Cholesterol and triglycerides serve as key biomarkers of lipid metabolism, with fluctuations in their levels reflecting metabolic activity and lipid homeostasis in animals [26]. This study revealed that triglyceride levels in the sodium butyrate group were significantly higher than those in the control group, aligning with the findings of Chen et al. [27], who reported that sodium butyrate enhances triglyceride synthesis by upregulating the enzymes involved in fatty acid biosynthesis. Additionally, total cholesterol levels were significantly elevated in the sodium acetate group compared to the control, possibly due to its regulatory influence on hepatic cholesterol synthesis and fatty acid metabolism. Previous studies suggest that sodium acetate promotes lipid metabolism by enhancing cholesterol biosynthesis, further supporting its role in lipid homeostasis [28]. Furthermore, total bilirubin levels were significantly higher in the sodium acetate group than in the control. Given that bilirubin is a metabolic byproduct of hemoglobin degradation following erythrocyte turnover [29], its increased concentration may be associated with enhanced hepatic metabolic function and bile acid secretion. Sodium acetate likely contributes to elevated bilirubin levels by modulating hepatic lipid metabolism and promoting bile acid synthesis, further highlighting its metabolic regulatory effects.

Total protein, albumin, and globulin serve as critical biochemical markers for assessing nitrogen metabolism and immune function. In this study, mares given sodium acetate and sodium butyrate exhibited an upward trend in these protein levels compared to the control group, suggesting that SCFAs may enhance protein synthesis and immune function by modulating nitrogen metabolism. This observation aligns with findings by Wang Yaling [5], who reported that dietary sodium acetate addition in dairy goats significantly increased blood total protein, albumin, and globulin concentrations, thereby improving nitrogen metabolism and immune function. Additionally, urea levels in the sodium butyrate group were significantly higher than in the sodium acetate and control groups, a result consistent with the findings of Tan [30], who demonstrated that dietary sodium acetate addition in perinatal dairy cows significantly elevated plasma urea levels, promoting protein catabolism and nitrogen excretion. These findings further support the regulatory role of SCFAs in nitrogen metabolism and protein turnover.

The contents of total protein, albumin, and globulin are important biochemical indicators for evaluating the nitrogen metabolism level of the body and are closely related to the body’s immune function. In this experiment, the contents of total protein, albumin, and globulin in the blood of mares supplemented with sodium acetate and sodium butyrate showed a certain upward trend compared with the control group. This result indicates that short-chain fatty acids may promote protein synthesis and improve immune function by regulating nitrogen metabolism. This finding is consistent with the research results of Wang Yaling [5], who found that adding sodium acetate to the basic diet of dairy goats could significantly increase the levels of total protein, albumin, and globulin in the blood, thereby improving nitrogen metabolism and immune function. In addition, this experiment also found that the urea content in the sodium butyrate group was significantly higher than that in the sodium acetate group and the control group. This result is consistent with the research results of Tan [30], who pointed out that adding sodium acetate in the diet of perinatal dairy cows could significantly increase the urea level in plasma, further promoting protein catabolism and nitrogen excretion.

Flavor is a comprehensive manifestation of the intrinsic quality of dairy products and an important part of the sensory evaluation of fresh milk [31]. Corresponding to human olfaction, taste, and touch, the flavor characteristics of milk can be analyzed from the dimensions of odor, taste, and texture [32]. Volatile compounds in milk are the main manifestation of its flavor. Volatile compounds are not only the main source of flavor but also the main substances that produce the odor of milk. Only some volatile compounds in raw milk are helpful for flavor formation [33]. The main volatile compounds in raw milk include terpenoids, acids (such as C4-C12 fatty acids), ketones (such as methyl ketones), aldehydes (such as nonenal, heptenal), esters (such as γ-lactone), phenols (such as *p*-cresol), and sulfides (such as dimethyl sulfide) [34]. Milk flavor is closely related to many factors, such as breed, diet, and feeding management [35]. Currently, diet is considered the most important and sensitive factor affecting the flavor of milk and dairy products. Nutrients such as carbohydrates, fats, and proteins in the diet can affect the flavor substances in milk through different pathways, thereby changing the flavor of milk [36]. Tu Xiaoting et al. [37] found that after short-chain fatty acids are ingested by the body, some are absorbed through the intestine and enter the mitochondrial citric acid cycle for energy supply, while others reach the liver through the portal vein and are ultimately converted into acetyl-CoA through β-oxidation in the mitochondria as the substrate for fatty acid synthesis in liver cells or participate in other metabolic pathways. Acetyl-CoA is an important intermediate product in biochemical pathways such as fatty acid synthesis and cholesterol synthesis. Through a series of enzymatic reactions, acetyl-CoA can participate in the synthesis of esters, acids, ketones, aldehydes, and other substances [38]. Further, Han Yaping et al. [39] found that substances such as alcohols, aldehydes, acids, esters, and ketones, which are the main volatile flavor components in milk, have a significant impact on the flavor of milk. Xue Haiyan et al. [40] identified 56 volatile substances in full-fat cow milk powder and 63 volatile substances in full-fat goat milk powder, including acids, alcohols, carbonyls, esters, aromatic compounds, and heterocyclic compounds. The results of this experiment indicate that a total of 86 volatile components were identified in the three groups of samples, including 16 esters, 15 alcohols, 15 ketones, three acids, 18 aldehydes, and nine others. Combining the GC-IMS three-dimensional, two-dimensional, difference, and fingerprint spectra of the volatile components in mare milk showed that the volatile substances in the control group, sodium acetate group, and sodium butyrate group have similar composition, but their signal intensities are different. The types of volatile substances in the sodium acetate group and sodium butyrate group are similar, and both are significantly higher than those in the control group, with the sodium butyrate group having the highest concentration. This indicates that supplementing mares with sodium acetate and sodium butyrate can increase the types and concentrations of volatile substances in mare milk compared to the control group. The rich volatile compounds in milk can provide a more diverse taste experience. Compared to the control group, the characteristic peak signals of various substances in mare milk after addition of sodium acetate were all enhanced, mainly concentrated in 2,3-diethylpyrazine, 2-nonanone, and heptanal. Different from the sodium acetate group, the characteristic peak signals of various substances in mare milk after addition of sodium butyrate were all enhanced, especially in aldehyde and ketone compounds, mainly concentrated in 2-heptenal, trans-2-octenal, 2-hexenal, trans-2-pentenal, 1-penten-3-one, and 3-octanone. Aldehydes and ketones are key compounds involved in regulating the flavor of dairy products and play a decisive role in the formation of dairy product flavor [41].

The unique flavor characteristics of milk are derived from the combined effects of various flavor substances, which are mainly composed of volatile organic compounds such as free fatty acids, alcohols, esters, and lactones [42]. Among them, acid compounds mainly come from the decomposition process of fats and biological metabolic pathways such as microbial fermentation. Acetic acid gives dairy products a vinegar-like taste and a slightly irritating odor. In this experiment, after supplementing mares with sodium butyrate compared to the control group, the relative content of acetic acid in mare milk was significantly reduced, further improving the flavor of mare milk. The reduction in the relative content of acetic acid may be due to the addition of short-chain fatty acids, which increased the energy level of the body, promoting more acetic acid to participate in energy metabolism and milk fat synthesis, thereby reducing the relative content of acetic acid in mare milk [43].

Ester compounds are an important component of the neutral volatile components in milk, formed by the esterification of short-chain fatty acids and alcohols. These substances emit pleasant fruit and floral aromas and are important sources of volatile flavor substances in milk [44]. Moio et al. [45] found that ethyl butyrate is a key flavor substance in fresh cow’s milk, goat’s milk, and sheep’s milk, with a unique fresh fruit aroma, which is crucial for improving the flavor of milk. In addition, ethyl L-lactate is also an important flavor substance in milk and food, with a slight fruity and wine-like odor, enhancing consumers’ olfactory experience of dairy products. This experiment found that after adding sodium acetate and sodium butyrate, the relative contents of ethyl butyrate and ethyl L-lactate in mare milk were significantly increased, improving the flavor and quality of mare milk. On the one hand, short-chain fatty acids can increase the activity of enzymes related to the synthesis of ester compounds by regulating the inflammatory response process of the body, promoting the synthesis of esters [46]; on the other hand, short-chain fatty acids participate in the synthesis and metabolism of milk fat, enriching the free fatty acids in the mammary gland through de novo synthesis or elongation of carbon chains, thereby affecting the metabolism of flavor substances in milk [47]. Alcohols are mostly formed by the oxidation of polyunsaturated fatty acids and have a sweet and mellow flavor characteristic. They account for a large proportion of the volatile flavor substances in milk [48]. Ji Quan et al. [49] demonstrated that abundant alcohols and ketones are the key volatile substances that affect the flavor of yak milk and cow milk products. In this study, mare milk with sodium acetate and sodium butyrate was rich in 1-penten-3-ol, pentanol, butanol, and cis-2-penten-1-ol. Through aroma characteristic retrieval, it was found that 1-penten-3-ol has a buttery aroma, pentanol has a grassy and fruity aroma, n-butanol has an alcohol aroma and a unique mature fruit aroma, and cis-2-penten-1-ol is a major component of fruity odors [50,51,52]. These flavor substances give mare milk a fresher flavor and a richer taste. The increase in alcohols may be related to the metabolism of short-chain fatty acids. After absorption in the intestine, short-chain fatty acids can be metabolized through gluconeogenesis. During gluconeogenesis, some intermediate products (such as pyruvic acid) may be further converted into alcohol compounds. Additionally, the increase in alcohols may be related to the potential ability of certain intestinal microorganisms to convert short-chain fatty acids into alcohols [53]. These microorganisms may convert short-chain fatty acids into corresponding alcohol compounds through specific enzymatic reactions [54]. This speculation may partially explain the effect of adding sodium acetate and sodium butyrate on mare milk flavor, but further research is needed for verification.

Most aldehydes in milk are derived from fat oxidation [55], while ketones are mainly produced through the oxidation and decomposition of unsaturated fatty acids, thermal decomposition processes, and the degradation of amino acids [56]. Both aldehydes and ketones play important roles in flavor formation. Zhang Lin et al. [57] demonstrated that the peak area of ketone components in volatile substances of cow milk is greater than that of other volatile components. Bai Xi et al. [58] showed that donkey milk powder has a rich variety of volatile flavor compounds, with a total of 54 volatile substances identified, among which aldehydes account for eight types. Studies have shown that 2-hexenal has a sweet almond and grassy aroma, often described as a “Green” aroma type, and is commonly used as a flavor enhancer in food [59]. Hexanal has a green, fruity, woody, and oily and grassy aroma, along with an apple-like scent; trans-2-octenal has a strong floral and fruity aroma; and propanal has a strong fruity aroma, such as apple and pear, and makes a significant contribution to the overall aroma of food. Gu Saiqi et al. [60] demonstrated that ketones have a certain effect in reducing fishy odors. For example, 3-pentanone is a characteristic flavor component of fresh fruits [61], 2-methyltetrahydrofuran-3-one has a sweet, nutty, and creamy aroma [62], 1-penten-3-one belongs to the fruity type of compounds [63], and 3-octanone as a volatile flavor substance can contribute a rich cheese-like aroma [64]. In this study, the relative contents of aldehyde flavor substances such as 2-hexenal, hexanal, 2-heptenal, heptaldehyde-M, pentanal, and 3-methylbutanal in mare milk with added sodium acetate and sodium butyrate were significantly increased; the relative contents of ketone flavor substances such as 2-methyltetrahydrofuran-3-one, 1-penten-3-one, 3-pentanone, and 3-octanone were all extremely significantly higher, and sodium butyrate addition significantly increased the ketone flavor substances in mare milk. Additionally, the relative content of 2-acetylpyridine in milk increased extremely significantly with the addition of sodium butyrate. 2-Acetylpyridine has a popcorn aroma, a prominent nutty and sweet aroma, and a fruity aroma [65], and is one of the key components for enhancing the flavor of mare milk. In addition, the relative content of dimethyl sulfide was significantly or extremely significantly higher than that of the control group. Dimethyl sulfide is not only a key aroma component of cooked garlic but also integrates flavors such as cabbage, vegetables, seafood, and cream, significantly enhancing the flavor of dairy products [66,67]. In this study, adding sodium acetate and sodium butyrate to the mares’ diet significantly increased the content of dimethyl sulfide and improved the flavor of the mare’s milk. In summary, after adding sodium acetate and sodium butyrate to the diet of mares, the relative content of aldehydes and ketones in the mare’s milk was increased, thereby generating aldehydes and ketones with different flavor characteristics, especially in the sodium butyrate group, where the effect was more significant.

## 5. Conclusions

This study systematically assessed the impact of sodium acetate and sodium butyrate on milk composition, blood biochemical parameters, and volatile flavor compounds in mare milk. The results demonstrated that the addition of sodium acetate and sodium butyrate significantly increased milk fat content in lactating mares while exerting minimal influence on milk protein and lactose levels. Blood biochemical analysis revealed distinct metabolic effects: sodium butyrate addition led to significant elevation of urea and triglyceride levels, whereas sodium acetate significantly increased glucose and total bilirubin concentrations, indicating differential roles in lipid metabolism and energy utilization. GC-IMS analysis further revealed that sodium acetate and sodium butyrate supplementation markedly enhanced the variety and concentration of volatile compounds in mare milk, particularly esters, aldehydes, and ketones, which are crucial for improving flavor complexity.

Given that the experiment was conducted under uniform grazing conditions with Yili mares of the same breed, growth stage, and lactation characteristics, it is reasonable to attribute the observed flavor modifications primarily to additive inclusion rather than environmental factors or breed differences. Future research should further explore the effects of various additives on optimizing mare milk flavor and investigate potential interactions between additives and other milk quality determinants under controlled conditions. A deeper understanding of these mechanisms will facilitate the development of improved mare milk products with enhanced sensory attributes.

In conclusion, sodium acetate and sodium butyrate improve milk flavor by modulating milk composition and metabolic profiles, providing a scientific foundation for enhancing the quality of mare milk products.

## Figures and Tables

**Table 1:** Nutrients in pasture ①.

Items	June	July	August	September
DM (%)	89.75	92.82	91.56	88.93
OM (%)	91.80	93.41	92.63	90.97
GE (MJ/kg)	17.43	18.14	17.64	16.98
CP (%)	11.36	10.98	10.12	9.64
EE (%)	2.70	2.80	2.77	2.66
CF (%)	35.57	36.46	36.75	37.84
Ca (%)	0.75	0.80	0.85	0.90
*p* (%)	0.14	0.17	0.15	0.14

Note: ① The nutrient content represents the actual measured value.

**Table 2:** Effects of sodium acetate and sodium butyrate addition on milk composition of Yili mares.

Item	Control Group	Sodium Acetate Group	Sodium Butyrate Group
Milk fat (%)	1.20 ± 0.37 ^Bb^	1.67 ± 0.25 ^AaB^	1.80 ± 0.19 ^Aa^
Milk protein (%)	1.63 ± 0.18	1.80 ± 0.12	1.70 ± 0.13
Lactose (%)	6.12 ± 0.25	6.37 ± 0.20	6.31 ± 0.18
Non-fat solids (%)	8.53 ± 0.46	8.98 ± 0.34	8.88 ± 0.37

Note: Different lowercase letters indicate significant differences (*p* < 0.05), while different uppercase letters denote highly significant differences (*p* < 0.01).

**Table 3:** Effects of sodium acetate and sodium butyrate addition on blood biochemical indices of Yili mares.

Item	Control Group	Sodium Acetate Group	Sodium Butyrate Group
Total protein (g/L)	60.75 ± 4.24	62.32 ± 3.09	63.78 ± 4.94
Albumin (g/L)	26.60 ± 1.34	27.21 ± 1.58	26.45 ± 0.96
Globulin (g/L)	34.15 ± 4.62	35.11 ± 2.76	37.33 ± 5.59
Urea (μmol/L)	6.86 ± 0.63 ^Bb^	7.06 ± 0.36 ^Bb^	8.47 ± 0.34 ^Aa^
Glucose (mmol/L)	1.54 ± 0.33 ^Bb^	2.93 ± 0.30 ^Aa^	1.93 ± 1.24 ^ABb^
Triglycerides (mmol/L)	0.29 ± 0.02 ^AaB^	0.17 ± 0.04 ^Bb^	0.36 ± 0.12 ^Aa^
Total bilirubin (mmol/L)	5.28 ± 0.34 ^Bb^	7.71 ± 1.40 ^Aa^	6.31 ± 0.77 ^ABb^
Direct bilirubin (μmol/L)	5.25 ± 0.17	6.10 ± 1.44	5.78 ± 0.20
Total cholesterol (μmol/L)	1.55 ± 0.09 ^AaB^	1.67 ± 0.18 ^Aa^	1.40 ± 0.08 ^Bb^

Note: Different lowercase letters indicate significant differences (*p* < 0.05), while different uppercase letters denote highly significant differences (*p* < 0.01).

**Table 4:** Qualitative analysis of volatile acid ions in ionic migration spectra.

Compound		Retention Time (Rt/s)	Relative Migration Time (Dt/ms)	Relative Retention Index (RI)	Relative Content (%)		
Name	Molecular Formula				Control Group	Sodium Acetate Group	Sodium Butyrate Group
Butanoic acid	C_4_H_8_O_2_	1653.709	1.16384	1667.9	0.53 ± 0.29	0.32 ± 0.04	0.34 ± 0.06
Acetic acid-M	C_2_H_4_O_2_	1155.249	1.06381	1495	5.72 ± 1.16 Aa	4.79 ± 0.48 AaBb	4.22 ± 0.57 Bb
Acetic acid-D	C_2_H_4_O_2_	1158.013	1.16514	1496.1	0.34 ± 0.14 Aa	0.16 ± 0.03 Bb	0.16 ± 0.03 Bb

Note: All relative content values are calculated as percentages (%) using peak volume normalization, representing the relative proportion of each volatile compound in the sample. Different lowercase letters indicate significant differences (*p* < 0.05), while different uppercase letters denote highly significant differences (*p* < 0.01).

**Table 5:** Qualitative analysis of volatile ester ions in gaseous ion migration spectra.

Compound		Retention Time (Rt/s)	Relative Migration Time (Dt/ms)	Relative Retention Index (RI)	Relative Content (%)		
Name	Molecular Formula				Control Group	Sodium Acetate Group	Sodium Butyrate Group
Butanoic acid ethyl ester-M	C_7_H_12_O_4_	1463.348	1.24302	1608.9	2.89 ± 1.09	2.39 ± 0.36	2.12 ± 0.25
Diethyl propanedioate	C_5_H_10_O_2_S	1065.004	1.1769	1455.7	4.04 ± 2.14 a	2.45 ± 0.39 b	1.96 ± 0.22 b
Ethyl (methylthio) acetate	C_8_H_16_O_2_	682.589	1.34208	1245.7	0.07 ± 0.06	0.03 ± 0.01	0.05 ± 0.04
Butanoic acid ethyl ester-D	C_6_H_12_O_2_	382.371	1.20978	1046.2	0.56 ± 0.39 a	0.19 ± 0.05 b	0.31 ± 0.15 ab
Isovaleric acid, methyl ester	C_6_H_12_O_2_	382.611	1.55631	1046.4	0.15 ± 0.14	0.04 ± 0.01	0.05 ± 0.02
Ethyl acrylate	C_6_H_12_O_2_	366.296	1.20335	1030.7	0.15 ± 0.05	0.13 ± 0.02	0.17 ± 0.03
Butanoic acid, methyl ester	C_5_H_8_O_2_	352.861	1.12288	1017.1	0.11 ± 0.05	0.11 ± 0.03	0.10 ± 0.01
Ethyl propanoate-M	C_5_H_10_O_2_	337.746	1.15078	1001.3	0.90 ± 0.19	0.72 ± 0.13	0.78 ± 0.22
Ethyl 2-methylpropionate	C_5_H_10_O_2_	313.754	1.1497	965.9	0.27 ± 0.26	0.11 ± 0.07	0.14 ± 0.07
Ethyl propanoate-D	C_5_H_10_O_2_	313.514	1.44903	965.6	0.13 ± 0.11	0.09 ± 0.01	0.09 ± 0.01
Acetic acid ethyl ester-M	C_6_H_12_O_2_	318.553	1.19905	973.5	0.06 ± 0.06	0.02 ± 0.01	0.05 ± 0.03
Acetic acid ethyl ester-D	C_4_H_8_O_2_	269.979	1.09723	891.3	1.03 ± 0.40	0.82 ± 0.34	1.18 ± 0.55
Ethyl formate	C_4_H_8_O_2_	270.184	1.3362	891.7	0.56 ± 0.52	0.26 ± 0.24	0.62 ± 0.62
Ethyl (S)-(-)-lactate	C_3_H_6_O_2_	231.546	1.072	815	2.08 ± 0.54	2.47 ± 0.25	2.42 ± 0.27
Ethyl caproate	C_5_H_10_O_3_	231.354	1.14638	814.5	3.68 ± 1.75 b	6.28 ± 1.79 a	5.18 ± 1.58 ab
Butanoic acid, hexyl ester	C_10_H_20_O_2_	946.027	1.48773	1398.6	0.22 ± 0.04	0.30 ± 0.10	0.28 ± 0.14

Note: Different lowercase letters indicate significant differences (*p* < 0.05).

**Table 6:** Qualitative analysis of volatile alcohol ions in ionic migration spectra.

Compound		Retention Time (Rt/s)	Relative Migration Time (Dt/ms)	Relative Retention Index (RI)	Relative Content (%)		
Name	Molecular Formula				Control Group	Sodium Acetate Group	Sodium Butyrate Group
1-Penten-3-ol	C_5_H_12_O	711.399	1.25693	1264	0.92 ± 0.26 Bb	1.45 ± 0.28 Aa	1.14 ± 0.10 ABb
1-Pentanol-M	C_5_H_12_O	711.399	1.51982	1264	0.14 ± 0.04 Bb	0.26 ± 0.08 Aa	0.18 ± 0.02 ABb
1-Pentanol-D	C_5_H_12_O	644.479	1.24834	1220.4	0.62 ± 0.59	0.27 ± 0.07	0.59 ± 0.49
1-Butanol, 3-methyl-M	C_5_H_12_O	645.665	1.49542	1221.2	0.16 ± 0.15	0.09 ± 0.01	0.14 ± 0.08
1-Butanol	C_5_H_10_O	561.017	0.93834	1169.8	0.90 ± 0.17 Bb	1.13 ± 0.19 Bb	2.14 ± 0.69 Aa
1-Propanol, 2-methyl-M	C_4_H_10_O	530.3	1.184	1153	0.38 ± 0.26	0.24 ± 0.06	0.28 ± 0.02
1-Propanol, 2-methyl-D	C_4_H_10_O	449.613	1.17727	1103.5	0.42 ± 0.38	0.18 ± 0.08	0.25 ± 0.13
1-Propanol	C_4_H_10_O	447.779	1.36741	1102.3	0.03 ± 0.02	0.02 ± 0.00	0.02 ± 0.00
1-Butanol, 3-methyl-D	C_3_H_8_O	384.051	1.1143	1047.8	0.73 ± 0.37	0.62 ± 0.12	1.01 ± 0.37
2-Butanol	C_4_H_10_O	367.736	1.15078	1032.1	0.13 ± 0.06	0.10 ± 0.02	0.26 ± 0.16
Ethanol	C_2_H_6_O	295.28	1.12503	935.8	8.00 ± 2.60 b	6.33 ± 2.76 b	9.34 ± 2.51 a
2-Propanol-D	C_4_H_10_O_2_	266.491	1.12789	884.8	0.31 ± 0.14 b	0.50 ± 0.16 a	0.37 ± 0.10 ab
1,3-Butanediol	C_3_H_8_O	289.264	1.09308	925.6	0.30 ± 0.11	0.26 ± 0.04	0.28 ± 0.04
2-Propanol-M	C_3_H_8_O	289.091	1.21996	925.3	0.40 ± 0.25	0.31 ± 0.05	0.44 ± 0.04
(Z)-2-Pentenol	C_5_H_10_O	825.342	0.93905	1332.8	0.23 ± 0.04 Bb	0.26 ± 0.03 Bb	0.34 ± 0.03 Aa

Note: Different lowercase letters indicate significant differences (*p* < 0.05), while different uppercase letters denote highly significant differences (*p* < 0.01).

**Table 7:** Qualitative analysis of volatile aldehyde ions in ionic migration spectra.

Compound		Retention Time (Rt/s)	Relative Migration Time (Dt/ms)	Relative Retention Index (RI)	Relative Content (%)		
Name	Molecular Formula				Control Group	Sodium Acetate Group	Sodium Butyrate Group
2-Hexenal-M	C_8_H_14_O	1021.437	1.33782	1435.6	0.10 ± 0.03 Bb	0.13 ± 0.02 Bb	0.28 ± 0.09 Aa
1-hexanal-M	C_7_H_12_O	816.859	1.25693	1327.8	0.22 ± 0.09 Bb	0.33 ± 0.10 Bb	1.05 ± 0.44 Aa
(E)-2-Heptenal-M	C_7_H_12_O	816.859	1.66744	1327.8	0.19 ± 0.04 Bb	0.22 ± 0.02 Bb	0.31 ± 0.07 Aa
(E)-2-Heptenal-D	C_6_H_10_O	663.822	1.18211	1233.5	0.27 ± 0.09 Bb	0.43 ± 0.20 AaBb	0.57 ± 0.15 Aa
Heptaldehyde-M	C_6_H_10_O	663.65	1.51378	1233.3	0.02 ± 0.00 b	0.03 ± 0.02 ab	0.04 ± 0.02 a
1-hexanal-D	C_7_H_14_O	608.278	1.34857	1194.9	0.74 ± 0.32	1.11 ± 0.63	0.83 ± 0.33
n-Pentanal-M	C_7_H_14_O	608.875	1.69169	1195.4	0.10 ± 0.07	0.14 ± 0.11	0.07 ± 0.03
n-Pentanal-D	C_5_H_8_O	513.338	1.10829	1143.2	0.63 ± 0.20 b	0.86 ± 0.40 ab	1.19 ± 0.59 a
3-Methylbutanal-M	C_5_H_8_O	512.879	1.36068	1143	0.10 ± 0.04 b	0.17 ± 0.14 ab	0.38 ± 0.32 a
3-Methylbutanal-D	C_6_H_12_O	439.527	1.27318	1096.6	0.55 ± 0.54	1.15 ± 0.76	0.72 ± 0.26
Acetaldehyde	C_6_H_12_O	439.068	1.5609	1096.2	0.10 ± 0.09	0.20 ± 0.18	0.10 ± 0.02
(E)-oct-2-enal	C_5_H_5_NO	360.059	1.09284	1024.4	0.26 ± 0.11	0.21 ± 0.05	0.23 ± 0.09
2-Hexenal-D	C_5_H_10_O	334.627	1.19584	997.9	1.24 ± 0.50 b	1.92 ± 0.43 a	1.40 ± 0.31 b
Heptaldehyde-D	C_5_H_10_O	335.107	1.42006	998.5	1.97 ± 0.85 ab	2.81 ± 1.21 a	1.58 ± 0.54 b
(E)-2-Pentenal-M	C_5_H_10_O	287.505	1.17149	922.5	0.55 ± 0.20	0.68 ± 0.16	0.58 ± 0.17
(E)-2-Pentenal-D	C_5_H_10_O	287.831	1.40366	923.1	0.75 ± 1.11	0.47 ± 0.17	0.39 ± 0.18
2-Formylpyrrole	C_2_H_4_O	189.997	0.95993	716.7	0.37 ± 0.12	0.32 ± 0.06	0.49 ± 0.22
Propanal	C_3_H_6_O	212.782	1.03634	773	1.78 ± 0.36 bc	2.21 ± 0.27 ABb	2.66 ± 0.23 Aa

Note: Different lowercase letters indicate significant differences (*p* < 0.05), while different uppercase letters denote highly significant differences (*p* < 0.01).

**Table 8:** Qualitative analysis of volatile ketone ions in gas-phase ionic migration spectra.

Compound		Retention Time (Rt/s)	Relative Migration Time (Dt/ms)	Relative Retention Index (RI)	Relative Content (%)		
Name	Molecular Formula				Control Group	Sodium Acetate Group	Sodium Butyrate Group
2-Nonanone-M	C_9_H_18_O	938.971	1.41062	1395	2.05 ± 1.41	2.16 ± 2.14	0.62 ± 0.10
Cyclohexanone	C_9_H_18_O	937.385	1.87775	1394.2	0.66 ± 0.35	0.81 ± 0.51	0.47 ± 0.05
2-Heptanone-M	C_5_H_8_O_2_	760.561	1.07291	1293.4	0.28 ± 0.06 Bb	0.30 ± 0.03 Bb	0.41 ± 0.08 Aa
2-Heptanone-D	C_6_H_10_O	758.182	1.1538	1292	0.15 ± 0.04	0.15 ± 0.04	0.20 ± 0.04
2-Nonanone-D	C_8_H_16_O	712.984	1.30951	1264.9	0.13 ± 0.02 Bb	0.17 ± 0.02 Bb	0.28 ± 0.10 Aa
2-methyl-3-ketotetrahydrofuran	C_7_H_14_O	601.75	1.25767	1190.8	4.52 ± 0.60 a	4.08 ± 1.59 ab	3.14 ± 0.17 b
1-Penten-3-one-D	C_7_H_14_O	601.75	1.63061	1190.8	5.08 ± 3.50	4.94 ± 5.45	1.35 ± 0.12
1-Penten-3-one-M	C_5_H_8_O	372.055	1.08211	1036.3	0.45 ± 0.17 Bb	0.85 ± 0.33 AaBb	1.23 ± 0.54 Aa
3-Pentanone-M	C_5_H_8_O	372.055	1.30848	1036.3	0.08 ± 0.03 Bb	0.21 ± 0.14 AaBb	0.60 ± 0.39 Aa
3-Pentanone-D	C_5_H_10_O	331.508	1.1143	993.3	1.17 ± 0.21 Bb	1.16 ± 0.16 Bb	1.55 ± 0.20 Aa
2-Butanone-M	C_5_H_10_O	332.708	1.3514	995.1	1.45 ± 0.40 Bb	1.61 ± 0.16 Bb	2.18 ± 0.60 Aa
2-Butanone-D	C_4_H_8_O	280.237	1.06445	909.8	2.05 ± 0.47	2.28 ± 0.15	2.25 ± 0.33
2-Propanone	C_4_H_8_O	279.622	1.24315	908.7	4.52 ± 1.17	4.47 ± 1.34	4.54 ± 0.45
3-Octanone	C_3_H_6_O	241.666	1.11626	836.2	14.98 ± 2.49	16.56 ± 1.78	16.01 ± 1.29
2-Hexanone	C_6_H_12_O	437.244	1.19279	1094.7	0.12 ± 0.01 Aa	0.13 ± 0.02 Aa	0.09 ± 0.01 Bb

Note: Different lowercase letters indicate significant differences (*p* < 0.05), while different uppercase letters denote highly significant differences (*p* < 0.01).

**Table 9:** Qualitative analysis of other volatile ions in gas-phase ionic migration spectra.

Compound		Retention Time (Rt/s)	Relative Migration Time (Dt/ms)	Relative Retention Index (RI)	Relative Content (%)		
Name	Molecular Formula				Control Group	Sodium Acetate Group	Sodium Butyrate Group
Allylsulfide-M	C_7_H_7_NO	1490.857	1.11382	1617.9	1.94 ± 1.06 Aa	0.97 ± 0.13 ABb	0.71 ± 0.06 Bb
Allylsulfide-D	C_8_H_12_N_2_	1064.255	1.22256	1455.4	0.70 ± 0.38	0.81 ± 0.83	0.27 ± 0.06
Dimethylsulfide	C_13_H_22_O	764.525	1.41669	1295.9	0.15 ± 0.06 Bb	0.20 ± 0.08 ABb	0.31 ± 0.09 Aa
1-(2-pyridinyl)ethanone	C_8_H_10_	507.836	1.078	1140	0.10 ± 0.03 Bb	0.14 ± 0.03 AaBb	0.18 ± 0.05 Aa
2,3-diethylpyrazine	C_6_H_10_S	505.544	1.12511	1138.6	1.12 ± 0.86	0.82 ± 0.28	1.10 ± 0.32
Theaspirane	C_6_H_10_S	503.71	1.32029	1137.6	0.31 ± 0.41	0.13 ± 0.05	0.22 ± 0.10
Triethylamine	C_6_H_15_N	181.381	0.94058	693.6	1.15 ± 0.36	1.45 ± 0.11	1.43 ± 0.20
Ethylbenzene	C_2_H_6_S	221.015	0.95484	791.8	2.72 ± 2.06	1.64 ± 0.10	2.04 ± 0.44
Pyrrolidine	C_4_H_9_N	360.469	1.04951	1024.8	3.58 ± 0.80	3.41 ± 0.56	3.42 ± 0.73

Note: Different lowercase letters indicate significant differences (*p* < 0.05), while different uppercase letters denote highly significant differences (*p* < 0.01).

## Data Availability

The data that support the findings of this study are available from the corresponding author upon reasonable request.
